# The characteristics of solid-phase substrate during the co-fermentation of lignite and straw

**DOI:** 10.1371/journal.pone.0280890

**Published:** 2023-01-26

**Authors:** Shufeng Zhao, Xile Liu, Hongyu Guo, Yidong Cai, Yongjun Wang, Daping Xia, Weizhong Zhao

**Affiliations:** 1 School of Energy Science and Engineering, Henan Polytechnic University, Jiaozuo, China; 2 School of Geoscience and Surveying Engineering, China University of Mining &Technology, Beijing, China; 3 Collaborative Innovation Center of Coalbed Methane and Shale Gas for Central Plains Economic Region, Jiaozuo, China; 4 School of Energy Resources, China University of Geosciences, Beijing, China; 5 School of Computer Science and Technology, Henan Polytechnic University, Jiaozuo, China; 6 Department of Environmental Engineering, Technical University of Denmark, Lyngby, Denmark; Universiti Teknologi Petronas: Universiti Teknologi PETRONAS, MALAYSIA

## Abstract

Co-fermentation of lignite and biomass has been considered as a new approach in achieving clean energy. Moreover, the study of the characteristics of solid phase in the synergistic degradation process is of great significance in revealing their synergistic relationship. Accordingly, in order to produce biogas, lignite, straw, and the mixture of the two were used as the substrates, the solid phase characteristics of which were analyzed before and after fermentation using modern analytical methods. The results revealed that the mixed fermentation of lignite and straw promoted the production of biomethane. Moreover, the ratios of C/O and C/H were found to be complementary in the co-fermentation process. Furthermore, while the relative content of C-C/C-H bonds was observed to be significantly decreased, the aromatics degree of lignite was weakened. Also, while the degree of branching increased, there found to be an increase in the content of cellulose amorphous zone, which, consequently, led to an increase in the crystallinity index of the wheat straw. Hence, the results provide a theoretical guidance for the efficient utilization of straw and lignite.

## 1. Introduction

Coal is a solid combustible mineral formed by ancient plants buried underground through complex biochemical and physical and chemical changes [1]. Organic matter in coal is a complex polymer organic compound, which is mainly composed of carbon, hydrogen, oxygen, nitrogen, sulfur and phosphorus [2]. When coal is used as fuel, the pollutants produced are important sources of long-term impact on atmospheric environmental quality. Crop straw refers to the residual roots, stems and leaves after crop harvest. China has a lot of straw production, reaching 900 million tons, but only a small part of the straw is fully utilized, the national straw utilization rate of less than 40% [3]. Due to the crystallinity of cellulose and the presence of lignin, it is difficult to degrade in the natural state, resulting in low utilization of crop straw, resulting in environmental pollution [4]. Effective utilization of straw can reduce the harm caused by straw burning, and has development potential in modern society with energy shortage and serious environmental problems.

Production of clean energy through the combined conversion of coal and straw has been considered as a useful approach in reducing the consumption of the traditional fossil energy and, accordingly, decreasing the resultant environmental pollutions [5]. Moreover, this strategy can remarkably improve the efficient use of resources. Currently, research studies on the combined utilization of coal and straw mainly focus on the application of such methods as co-pyrolysis, co-combustion, co-liquefaction and co-gasification [6–10]. Moreover, the co-thermal synergistic conversion of the two elements is promoted by high H/C, hydrogen-rich radicals, alkali metals, biomass char, lignin pyrolysis intermediates (such as phenoxy and benzyloxy), highly active pyrolysis gas [11, 12]. Yet, the difficulties and challenges in such conversion methods as pyrolysis, gasification, liquefaction. have also led clean energy not to be produced at the large-scale industrial levels. Hence, the investigation of a new combined pretreatment method is of high significance. In line with this, the anaerobic fermentation technology can be considered as a new conversion approach in the combined clean utilization of coal and straw, which can be converted to green energy under low energy consumption conditions.

In the anaerobic fermentation process, the stability and, accordingly, the efficiency of fermentation system can be enhanced through the co-fermentation of coal and straw [13, 14]. Moreover, an appropriate increase in the straw can promote the efficient conversion of high concentration lignite to biogenic methane [15]. The co-fermentation with coal of such straws as rice, corn, wheat, sweet sorghum, has also been shown to promote biomethane production [16]. In one study, Guo et al. showed the different promoting effects of corn straw on the conversion into biomethane of coal of different ranks. Moreover, the methanogenic archaeal community was found to change significantly when coal and corn straw were mixed to be fermented [17, 18]. Also, the obtained results by Zhang et al. corroborated the effect of mixed fermentation of coal and corn straw on the increase in the abundance of certain key enzyme genes (amino acid metabolism, energy metabolism, lipid metabolism), which consequently, increased the gas production [19].

In the process of anaerobic fermentation, the physical and chemical properties of coal and straw (such as element content, surface functional groups, porosity, microcrystalline structure.) are affected and changed by the microbial action to varying degrees [20]. At the same time, these changes also affect the microbial metabolism of the microorganisms in the anaerobic fermentation system. So, with regards to the gas production, the advantages of mixed fermentation is theoretically supported by the variation characteristics of solid substrates during the anaerobic fermentation. However, thus far, the investigation of the synergistic degradation characteristics of solid substrates has been understudied. Therefore, the present study aims at investigating the use of lignite and wheat straw as the substrates of mixed fermentation as well as the single substrate anaerobic fermentation. Moreover, the solid-phase degradation characteristics of lignite and wheat straw are analyzed during the methanogenesis. The obtained results are expected to provide a solid-phase substrate perspective for further revealing the synergistic mechanism of mixed fermentation of different substrates. Furthermore, the findings can also provide a reference for further joint, multi-level and clean utilization of degraded lignite and straw.

## 2. Materials and methods

### 2.1. Inoculum and substrate

Fresh lignite, obtained from Shengli Coalfield, Inner Mongolia, was used as the coal sample in the present study. To avoid oxidation, lignite was stored in a sealed tank after being transported to the laboratory. Also, the wheat straw was collected from Suqian City, Anhui Province. The wheat straw was cut to about 1 cm in length, and the lignite was crushed to 100–150 mesh. The straw and coal samples were sterilized before use to prevent exogenous microbial contamination. Anaerobic enrichment culture was applied to enrich the mixed methanogenic bacteria from fresh coal samples of various regions (Yima mining area, Henan Province, Liulin mining area, Shanxi Province, Wulantuga mining area, Inner Mongolia.). Furthermore, the same method used by Su et al. (2018) for the enrichment of the bacterial source was used in this research [21]. The proximate analysis of the coal samples is provided in Table 1.

**Table 1:** Proximate analysis of coal sample.

Sample	Source of sample	*M*_ad_	*A*_ad_	*V*_ad_	*F*_Cad_	*R*_o, ran_
Lignite	Shengli Coalfield, Inner Mongolia	12.56	12.01	34.76	40.64	0.57

Note: *M*, moisture; *A*, ash yield; *V*, volatile matter; *FC*, fixed carbon; ad, air-dry basis; *R*_o, ran_, vitrinite random reflectance

### 2.2. Batch experiment setup

Lignite, wheat straw, and a mixture of lignite and wheat straw were used as the substrates of the anaerobic fermentation. Prior to the fermentation, the bioreactor was sterilized at 121°C for 20 mins. As can be seen in Table 2, the lignite, straw and bacteria were mixed in the reactor and charged with nitrogen for 5 mins, subsequently. Next, they were sealed and placed in an electric incubator at 35°C for the anaerobic fermentation, Shanghai Jinghong electric constant temperature incubator (DNP-9272), the specific parameters: power supply voltage: 220 V-50 HZ, temperature fluctuation: ±0.5°C; studio size: 600*600*750 mm, temperature control range: RT+5–65°C. A graduated syringe was used to test the obtained gas and methane collected by a gas sampling bag (Agilent 7890 GC, Agilent Technologies Inc., Santa Clara, CA, USA).

**Table 2:** Fermentation substrate ratio of contrast experiment for biomethane production.

Substrate composition	Coal mass /g	Wheat straw /g	Bacterial source /mL
Blank group	0	0	200
Lignite	20	0	200
Wheat straw	0	6.25	200
Lignite+Wheat straw	20	6.25	200

### 2.3. Ultimate analysis

In the experiment, the solid phase characteristics of the coal sample and the wheat straw were analyzed before and after the biogas production in different fermentation systems. The number of the samples are shown in Table 4. Flash 2000 (Thermal Flasher Scientific, America) organic element analyzer was used to test and analyze the contents of carbon (Cd, dry basis), hydrogen (Hd, dry basis), nitrogen (Nd, dry basis), and the total sulfur (Sd, dry basis). In the CHNS mode, the carrier gases were helium and oxygen. Moreover, the flow rates of the helium and oxygen, the temperatures of the left furnace and the column, and the oxygen admission time were 140 mL/min, 250 mL/min, 950°C, 65°C, and 5 mins, respectively. On the other hand, in the O mode, the oxygen was turned off, helium flow rate was 100 mL/min, column temperature was 65°C, and the right furnace temperature was 1060°C.

### 2.4 Solid phase characteristic test

The XPS (PHI Quantera SXM, ULVAC-PHI, Japan) was used to determine the occurrence of carbon, nitrogen, oxygen and sulfur on the surface of the sample. The test conditions were as follows: a hemispherical energy analyzer and an Al target were used. Also, an X-ray beam spot of 200 μm, an incident angle of 45°, an analysis chamber vacuum of 1.0×10^−7^ Torr were utilized. The measured transmission energy of wide-spectrum scanning and fine-spectrum scanning was 100 eV and 30 eV, respectively. FTIR experiment was carried out using FTIR Vertex 80v instrument. The sample was scanned 32 times in a wavenumber range of 4000–400 cm^-1^ with a spectral resolution of 8 cm^−1^. Moreover, to characterize the carbon macromolecular skeleton of the coal samples, the ^13^C spectra of the samples were scanned using JNM-ECZ600R nuclear magnetic resonance instrument. The coal sample was crushed to 200 mesh and dried at 40°C for 12 h. The solid dual-resonance probe as well as the ZrO_2_ rotor with an outer diameter of 6 mm were also utilized. The magic angle spinning, the resonance frequency of the ^13^C detection core, and the sampling time were 6–7 kHz, 75.43 MHz, and 0.05 s, respectively. The chemical shift was calibrated with the standard HMB (hexamethylbenzene). In addition, the pulse width, the cycle delay time, and the scan were 4.2×10^−6^ s, 4 s, and 2000–4000 times, respectively. D8 ADVANCE diffractometer (Bruker-AXS, Karlsruhe, Germany) XRD was also used to analyze the crystallinity of wheat straw before and after degradation in the different fermentation systems. Additionally, a Cu/Kα radiation source with a wavelength of 1.5418 nm and a scanning voltage of 45 kV, a scan range of 5°-40°, a step width of 0.03°, and a scan rate of 10.0 s/step was utilized.

## 3. Results and discussion

### 3.1. Biogas production

**Fig 1:**
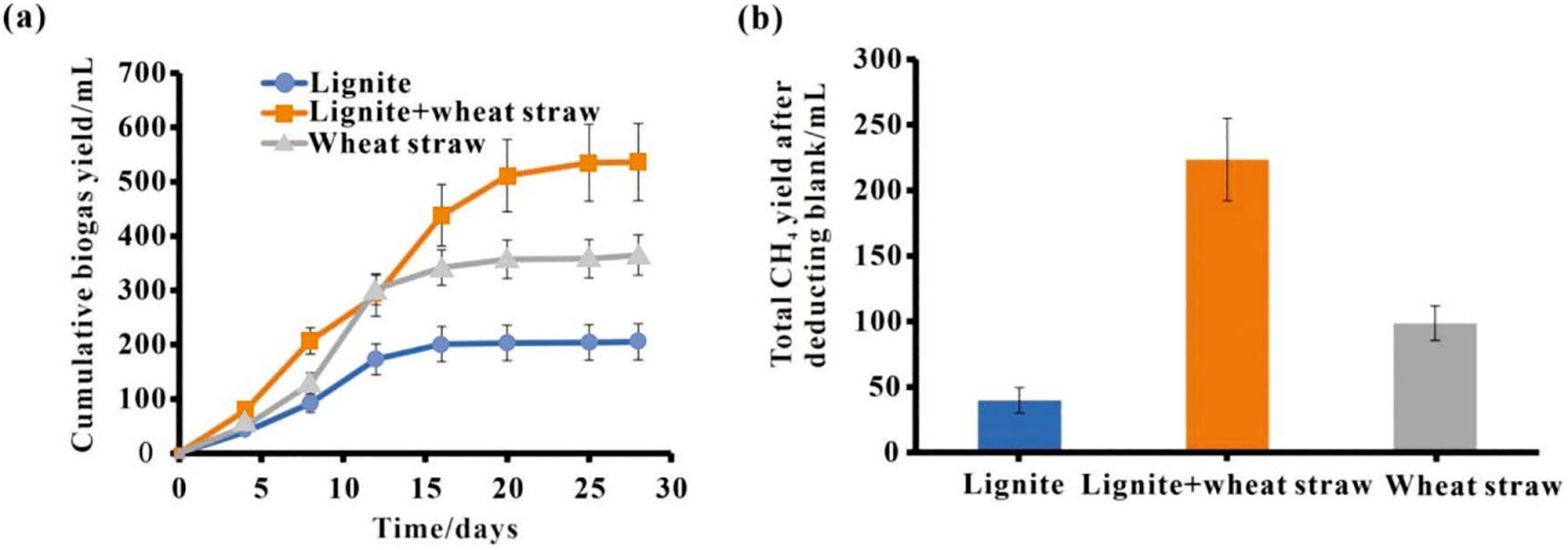
The cumulative biogas production (a) and total biomethane production (b) of different fermentation systems.

As can be seen in Table 3 and Fig 1(a), the cumulative gas production of the single lignite is 205.60 mL, minus by the blank control group (66.00 mL), the net cumulative gas production of which is 139.60 mL. The volumes were 365.00 mL and 299.00 mL for the cumulative gas production and the net cumulative gas production of the single wheat straw fermentation group, respectively. Moreover, they were found to be 536.70 mL and 470.70 mL for the lignite and wheat straw mixed fermentation group. So, the obtained results revealed an increase by 331.10 mL and 171.70 mL in the net cumulative gas production of the lignite and wheat straw mixed fermentation group, respectively. In other words, compared with the single lignite and the single wheat straw fermentation groups, gas production increased by 237.18% and 57.42%, respectively.

**Table 3:** Comparison of gas production of different substrate systems.

Substrate systems	Total gas production/mL	Methane production/mL
Blank	66.00	22.12
Lignite	205.60	61.84
Single Wheat straw	365.00	120.75
Lignite+Wheat straw	536.70	245.62

As can be observed in Fig 1(b), considering the total methane production as the index, the volume of methane production in the single lignite, the single wheat straw, and the mixed fermentation groups were 39.72 mL, 98.63 mL, and 223.50 mL, respectively (after subtracting the blank group). This is to say that, compared with the single lignite and the single wheat straw fermentation groups, the methane production of the mixed group increased by 462.69% and 126.60%, respectively. This is while, compared with the single lignite and the single wheat straw fermentation groups, the cumulative biomethane production increased by 61.19%. Therefore, the obvious effect of the co-fermentation of lignite and the wheat straw on the production of biomethane is supported.

### 3.2. Changes of element content before and after synergistic degradation

The elemental content of lignite and straw in each reaction system changed after the anaerobic fermentation differently. As can be seen in Table 4, compared with the original lignite (M0), the carbon and oxygen contents of the single lignite fermentation group (M1) were reduced by 0.53% and 0.94%, respectively. Likewise, the carbon and oxygen contents of the mixed fermentation group (M2) were reduced by 0.96% and 0.43%, respectively. Also, compared with the original wheat straw (G0), the content of these elements in the single wheat straw fermentation group (G1) increased by 1.13% and 1.87%, respectively. Similarly, there was an increase by 1.03% and 2.57% in the contents of carbon and oxygen in the mixed group (G2), respectively. Hence, while the contents of the carbon and oxygen in the coal decreased after the microbial degradation, those in the straw, as well as the hydrogen in the lignite and hydrogen in the straw increased.

**Table 4:** Changes of element content before and after degradation of lignite and straw.

Numbers	Samples	Source	*C*_d_/%	*H*_d_/%	*O*_d_/%	*C*/*H*	*C*/*O*
M0	Lignite	Original lignite	63.27	4.56	22.22	13.8750	2.8474
M1	Lignite	Single lignite fermentation group	62.74	4.60	21.28	13.6391	2.9483
M2	Lignite	Mixed fermentation group	62.31	4.71	21.79	13.2293	2.8596
G0	Wheat straw	Original wheat straw	46.22	6.03	38.70	7.6650	1.1943
G1	Wheat straw	Single wheat straw fermentation group	47.35	6.17	40.57	7.6742	1.1671
G2	Wheat straw	Mixed fermentation group	47.25	6.08	41.27	7.7714	1.1449

**Fig 2:**
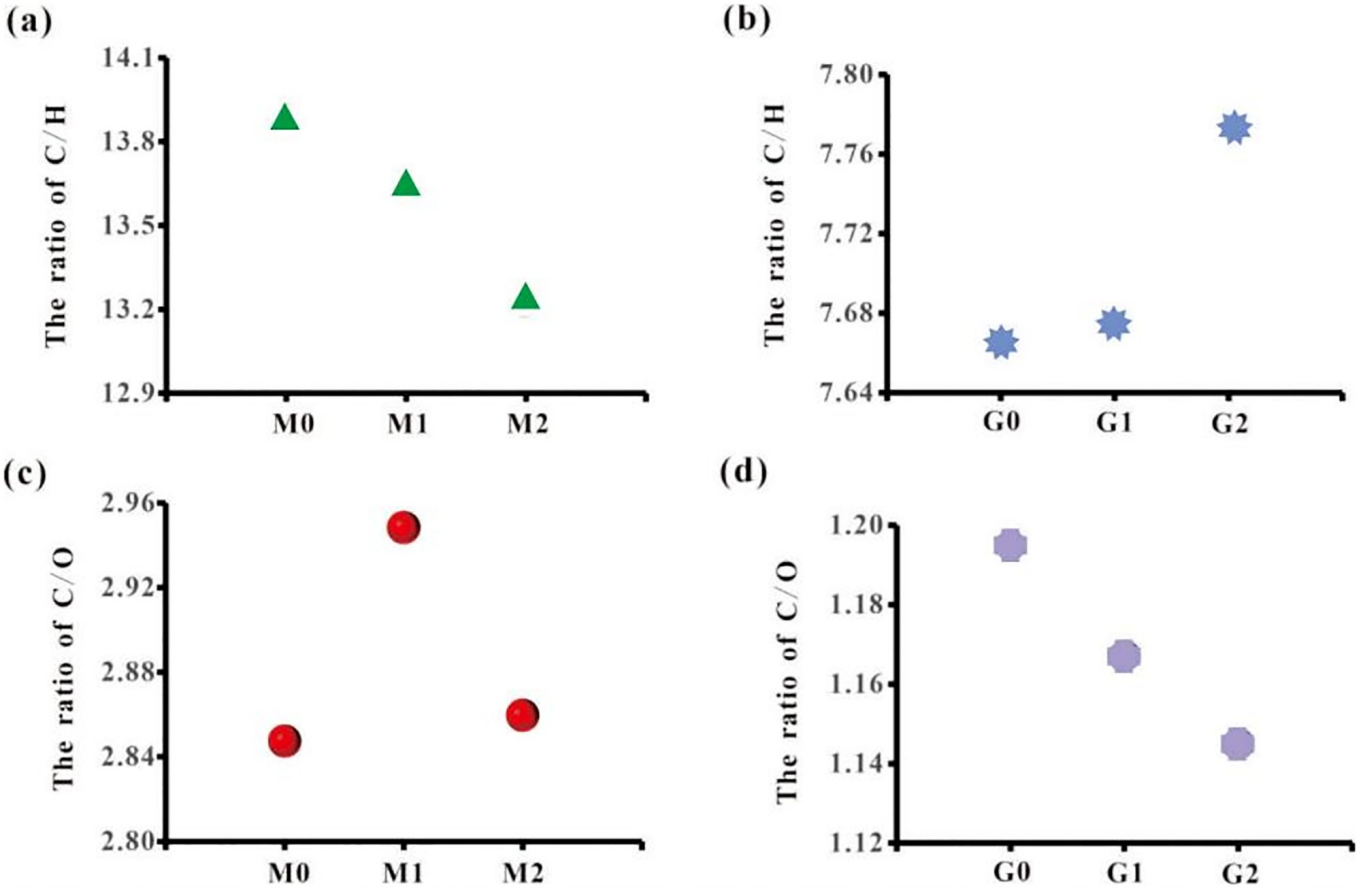
Changes in C/H ratio of lignite (a) and wheat straw (b); changes in C/O ratio of lignite (c) and wheat straw (d).

As shown in Fig 2(a), compared with M0, the C/H ratios of M1 and M2 decreased by 1.7% and 4.7%, respectively. Moreover, compared with G0, the C/H ratios of G1 and G2 increased by 0.12% and 1.39%, respectively (Fig 2(b)). Hence, while the C/H ratio in coal was found to have a decreasing trend, that of the straw had an increasing trend after the anaerobic fermentation. Furthermore, as can be observed in Fig 2(c), compared with M0, the C/O ratios of M1 and M2 increased by 4.08% and 0.43%, respectively. As compared with G0 (Fig 2(d)), the C/O ratios of G1 and G2 were found to decrease by 2.28% and 4.14%, respectively. Accordingly, while the C/O ratio in coal was found to have an increasing trend after the anaerobic fermentation, that of the straw had a decreasing trend.

The decreasing tendency of the C/H ratio in lignite after anaerobic fermentation as well as the increasing tendency of the C/O ratio are an indication of the relatively less consumption of hydrogen element in lignite than the carbon element. Moreover, it is an indication of the relatively more consumption of oxygen than carbon in the process of methane production. Furthermore, the increasing trend of the C/H ratio in the straw as well as the decreasing trend of the C/O ratio suggest the more consumption of hydrogen than carbon in the straw as well as the less consumption of the oxygen than carbon in the process of methane production. As can be observed, the changing trends of C/H and C/O ratios in coal and those of C/H and C/O ratios in straw constitute a complementary relationship in the process of methane production. This is to say that, they are found to be more conducive to fermentation gas production in the mixed fermentation process.

### 3.3 Variation characteristics of organic carbon forms on coal and straw surface

**Fig 3:**
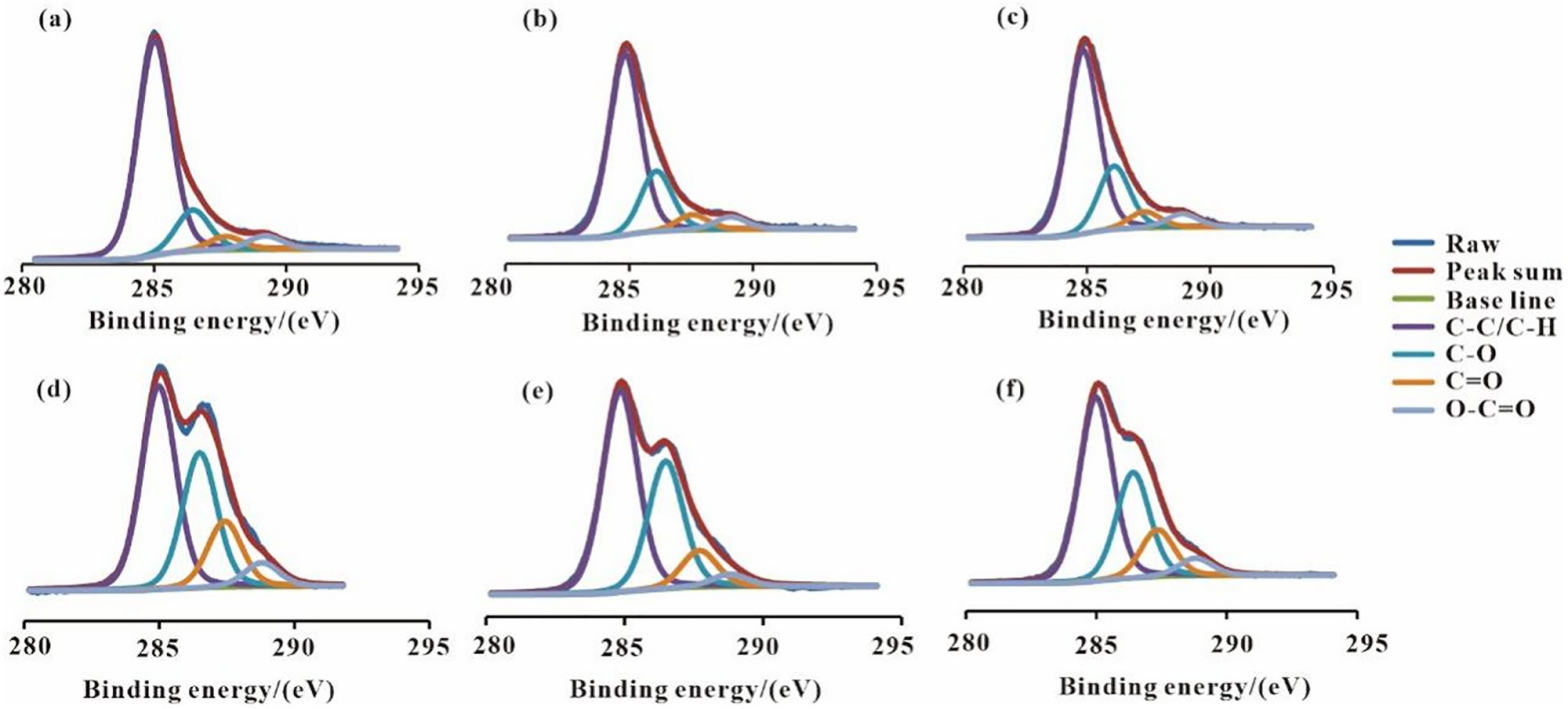
XPS fitting of different forms of organic carbon in M0 (a), M1 (b), M2 (c), G0 (d), G1 (e) and G2 (f).

XPS peak4.1 software was used to analyze the XPS data. Moreover, to fit the XPS spectra of organic carbon C1s, the Gaussian-Lorentzian (80%-20%) was applied. The obtained results revealed that the binding energy of the C-C/C-H, the C-O, the C = O/O-C-O, and the O-C = O bonds were 285.0±0.2 eV, 286.3±0.2 eV, 287.5±0.2 eV, and 289.0±0.2 eV, respectively. The full width at half maximum (FWHM) is 1.5 eV (Fig 3) [22, 23]. The fitting results are shown in Table 5.

**Table 5:** Relative content of organic carbon in different forms/%.

Sample numbers	C-C/C-H	C-O	C = O	O-C = O
M0	75.99	14.51	4.74	4.76
M1	67.23	22.38	5.70	4.68
M2	66.33	22.94	5.88	4.85
G0	53.42	33.75	9.65	3.18
G1	51.96	29.88	13.24	4.92
G2	47.55	31.58	15.36	5.51

As shown in Table 5, the relative content of C-C/C-H bonds on the surface of the coal sample was the highest. This was followed by C-O bonds. Moreover, the relative content of C = O and O-C = O bonds was found to be low. Compared with M0, the relative content of C-C/C-H bonds on the surface of M1 and M2 decreased by 8.76% and 9.66%, respectively. In addition, while the relative content of C-O bonds increased by 7.87% and 8.43%, that of C = O bonds increased by 0.96% and 1.14%, respectively. Furthermore, the further decrease in the relative content of C-C/C-H bond of the coal sample M2 in the mixed fermentation group is an indication of an increase in the degradation degree of the sample in the mixed group. At the same time, compared with G0, while the relative content of C-C/C-H and C-O bonds on the surface of G1 and G2 decreased, that of C = O and O-C = O bonds increased. As can be seen, while the relative content of C-C/C-H and C-O bonds on the surface of the wheat straw decreased after the anaerobic fermentation, that of C = O and O-C = O bonds increased. To summarize, the relative content of C-C/C-H bond and the oxygen-containing bond revealed a decreasing and an increasing trend after the biodegradation of lignite and straw, respectively. Moreover, further decrease in the relative content of C-C/C-H bond in the coal sample and straw in the mixed group was an indication of the degradation advantage of the mixed fermentation of lignite and straw.

### 3.4 Changes of infrared functional group content of lignite and straw

#### 3.4.1 Change of infrared functional group content of lignite

**Fig 4:**
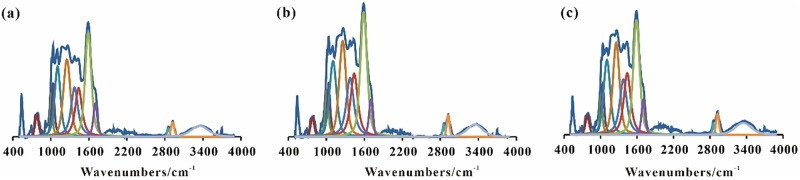
FTIR fitting of different functional groups in M0 (a), M1 (b), M2 (c).

The FTIR spectral absorption peaks of the coal samples were divided into three categories (Fig 4). Moreover, the aliphatic absorption peaks were 1380, 1435 and 3000–2800 cm^-1^. Also, while the absorption peaks of aromatic structure belonged to 900–700 and 1615–1585 cm^-1^, those of the oxygen-containing functional groups were 1300–1000, 1745–1630 and 3600–3200 cm^-1^, respectively.

The infrared test results can be used for the calculation of the infrared structural parameters of the coal samples. In line with this, *H*_al_/*H* and *f*_a_ represent the ratio of aliphatic hydrogen (*H*_al_) to the total hydrogen (*H*) and the apparent aromaticity, respectively. These parameters, i.e., *H*_al_/*H* and *f*_a_, were calculated by the application of the methods in the literature [24–27]. Moreover, the structural parameter ‘C’ of the oxygen-containing functional groups, representing the ratio of C = O and C = C functional groups, can be used to describe the maturity of the coal. Aliphatic structure parameter *A*(CH_2_)/*A*(CH_3_) and structure parameter ’C’ of oxygen-containing functional groups’ can be referenced [22].

The structural parameters of coal samples were calculated according to the equations in reference [24–27] (Table 6). Accordingly, the *H*_al_/*H*, *f*_a_, ‘*C*’, *A*(CH_2_)/*A*(CH_3_) structure parameters of M0 were 0.467, 0.9818, 0.171, 2.163, respectively. After the anaerobic fermentation, *H*_al_/*H* in the coal samples increased, indicating an increase in the content of the aliphatic hydrogen. On the other hand, the values of *f*_a_ and ‘*C*’ decreased, which was an indication of a decrease in the apparent aromaticity and maturity of the coal samples. Moreover, the *A*(CH_2_)/*A*(CH_3_) value decreases, which suggested a decrease and an increase in the long chain as well as the short chain of the fat in the coal samples, respectively. Consequently, it is suggested that the aromatization degree of lignite decreased with the microbial degradation. Furthermore, compared with M1, the *H*_al_/*H* value of M2 increased by 0.027. In addition, the *f*_a_, ‘*C*’ and the *A*(CH_2_)/*A*(CH_3_) values decreased by 0.0008, 0.001 and 0.053, respectively. Also, compared with the single lignite fermentation group, while the *H*_al_/*H* value of the mixed group was further increased, the *f*_a_, ‘*C*’ and *A*(CH_2_)/*A*(CH_3_) values were further decreased, indicating the greater degradation degree of the mixed fermentation group. Hence, the mixed fermentation of wheat straw and lignite was found to promote the further degradation of the lignite.

**Table 6:** Infrared structural characteristic parameters of coal samples.

Samples	*H*_al_/*H*	*f*_a_	‘*C*’	*A*(CH_2_)/*A*(CH_3_)
M0	0.467	0.9818	0.171	2.163
M1	0.533	0.9784	0.150	1.929
M2	0.560	0.9776	0.149	1.876

#### 3.4.2. Changes of infrared functional group content of wheat straw

**Fig 5:**
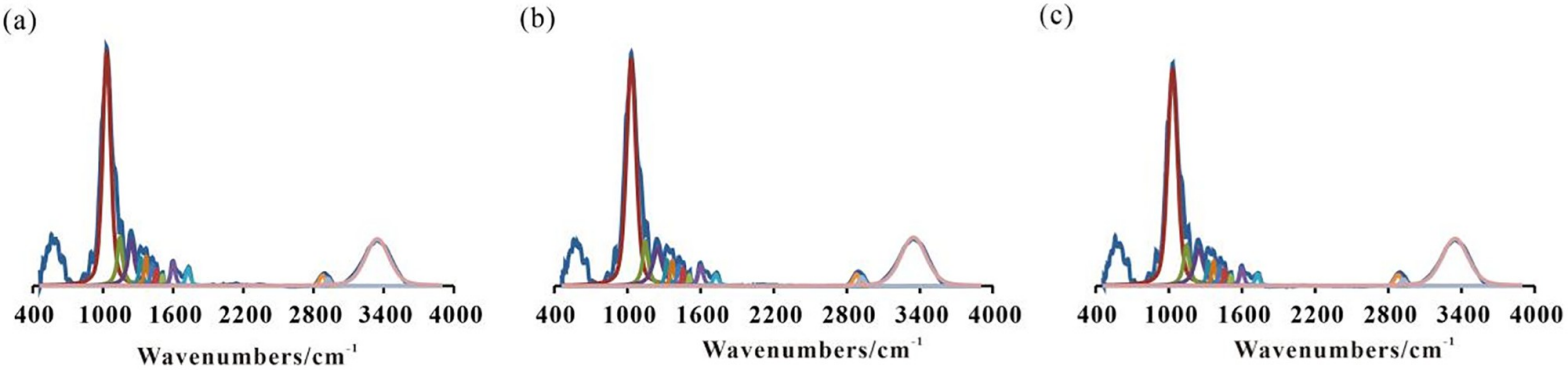
FTIR fitting of different functional groups in G0 (a), G1 (b) and G2 (c).

The fitting results of the infrared spectral functional groups of wheat straw are shown in Fig 5. the fitting peak positions of the infrared spectra of wheat straw (1–13) refer to [28, 29]. Moreover, the fitting results of the infrared spectra of wheat straw are shown in Table 7.

**Table 7:** Fitting results of infrared spectra of straw.

Samples	Relative content of each peak /%												
	1	2	3	4	5	6	7	8	9	10	11	12	13
G0	44.34	7.33	8.60	2.54	2.46	1.77	1.20	0.43	2.45	1.87	1.36	0.83	24.81
G1	45.32	6.63	9.02	2.31	2.15	1.68	1.19	0.50	2.33	1.45	1.42	1.07	24.92
G2	46.02	6.50	8.80	2.25	2.15	1.64	1.24	0.46	1.82	1.26	1.57	0.75	25.54

As can be observed in Fig 5, the C-O stretching vibration absorption peak of cellulose and hemicellulose in wheat straw (1061–1042 cm^-1^), the C-O-C antisymmetric stretching vibration peak of cellulose, hemicellulose and lignin, the C-O and amino acid C-N stretching vibrations in carbohydrates, the deformation vibration in the C-H plane of lignin guaiacol (G-type) ring (1162–1159 cm^-1^), and the C-O-C and C-O stretching vibration peaks in phenols, aromatic ethers and esters (1344–1253 cm^-1^). The relative content of stretching vibration absorption peak (3570–3050 cm^-1^) of hydroxyl compound -OH is higher. Moreover, compared with G0, the total relative content of methyl/methylene/methylene (peaks 5, 6, 11 and 12) in G1 and G2 decreased by 0.10% and 0.31%, respectively. Also, the relative content of C-O stretching vibration absorption peak (No. 1 peak) of cellulose and hemicellulose increased by 0.98% and 1.68%, respectively. The values were 0.11% and 0.73% for that of hydroxyl compound OH (No. 13 peak). Furthermore, a decrease by 0.54% and 1.24% were observed for the relative content of carbonyl (peaks 9 and 10), respectively. In addition, while the relative content of C-O-C of phenol, aromatic ether and ester (peaks 3 and 4) decreased by 0.19% in G1, it increased by 0.09% in G2. Hence, generally speaking, compared with the single straw fermentation, the relative content of the infrared functional groups of wheat straw significantly changed after degradation in the mixed group. Accordingly, this was an indication of the degradation of some cellulose and hemicellulose after the anaerobic fermentation. At the same time, the vibration peaks of methyl, methylene and methine were enhanced. Hence, the mixed fermentation was found to be beneficial to the further degradation of straw.

### 3.5 Changing characterization of carbon skeleton structure of lignite

**Fig 6:**
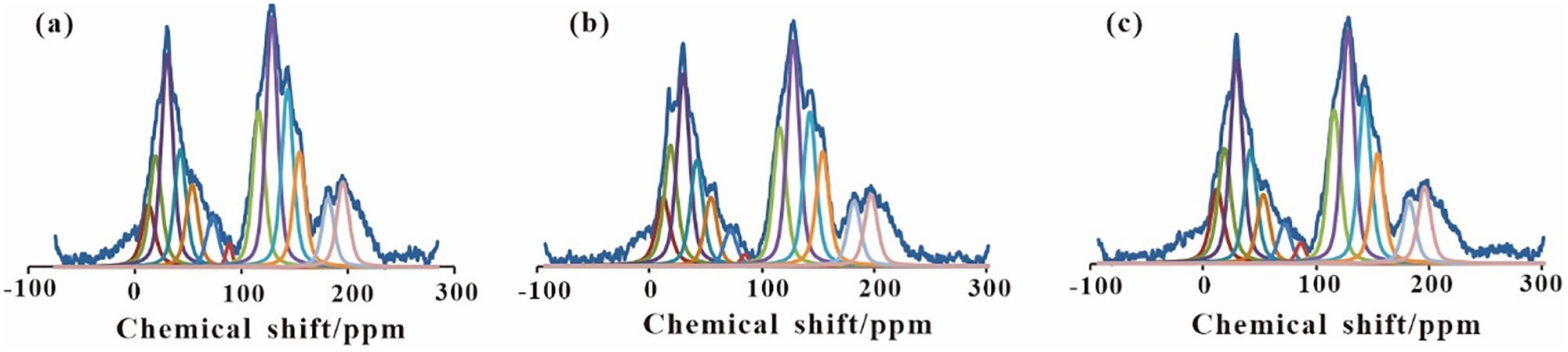
13C-NMR fitting of M0 (a), M1 (b), M2 (c).

As can be observed in Fig 6, ^13^C-NMR chemical shifts were divided into three regions: (1) the lipid carbon peak with a chemical shift of about 0–90 ppm; (2) the aromatic carbon peak with a chemical shift of about 100–165 ppm; and (3) the carbonyl carbon peak with a chemical shift of about 200–220 ppm. The NMR fitting peak positions of coal samples refer to References [30, 31]. The fitting results are shown in Table 8.

**Table 8:** Fitting results of NMR of lignite.

Sample numbers	Relative content of each peak/%												
	1	2	3	4	5	6	7	8	9	10	11	12	13
M0	4.78	7.91	13.85	7.82	5.54	3.18	0.80	10.31	15.99	11.77	7.80	4.55	5.70
M1	5.82	9.44	13.85	7.72	4.81	2.44	0.51	10.08	15.74	11.11	8.57	4.71	5.20
M2	5.80	8.69	13.93	7.96	4.80	2.55	1.05	10.52	15.72	11.54	7.74	4.34	5.36

According to the NMR spectra of the coal samples as well as the fitting results in Table 8, the rates of the aromatic and aliphatic carbons of M0 were found to be 45.87% and 34.36%, respectively. Moreover, compared with M0, while the aromatic carbon rate of M1 and M2 decreased by 0.35% and 0.37%, the aliphatic carbon rate increased by 1.02% and 1.47%, respectively. Furthermore, an increase by 1.02% and 1.04% of the relative content of terminal methyl (aliphatic methyl carbon-CH_3_) was observed. Also, the relative content of methyl on ring was found to be increased by 0.78% and 1.53%, respectively.

According to the obtained results of the branching degree L_γ_ (Table 9), compared with M0, the branching degree of M1 and M2 increased by 0.044 and 0.049, respectively, after the anaerobic fermentation. Moreover, the branching degree of lignite was observed to be increased after the fermentation, which is consistent with the results obtained from the infrared spectroscopy. Also, compared with the single lignite fermentation group, the branching degree of lignite in the mixed group was observed to be increased by 0.005. Hence, the branching degree of lignite was observed to be further increased in the mixed fermentation of lignite and straw, which in turn, aggravated the further degradation of the coal samples.

**Table 9:** The branched chain degree of lignite.

Sample numbers	Relative percentage of fat carbon/%				Branching degree *L*_γ_
	Terminal methyl	Methyl on the ring	Methylene, hypomethyl	Quaternary carbon	
M0	4.78	7.91	13.85	7.82	0.221
M1	5.80	8.69	13.93	7.96	0.265
M2	5.82	9.44	13.85	7.72	0.270

### 3.6. Changes in cellulose crystallinity

**Fig 7:**
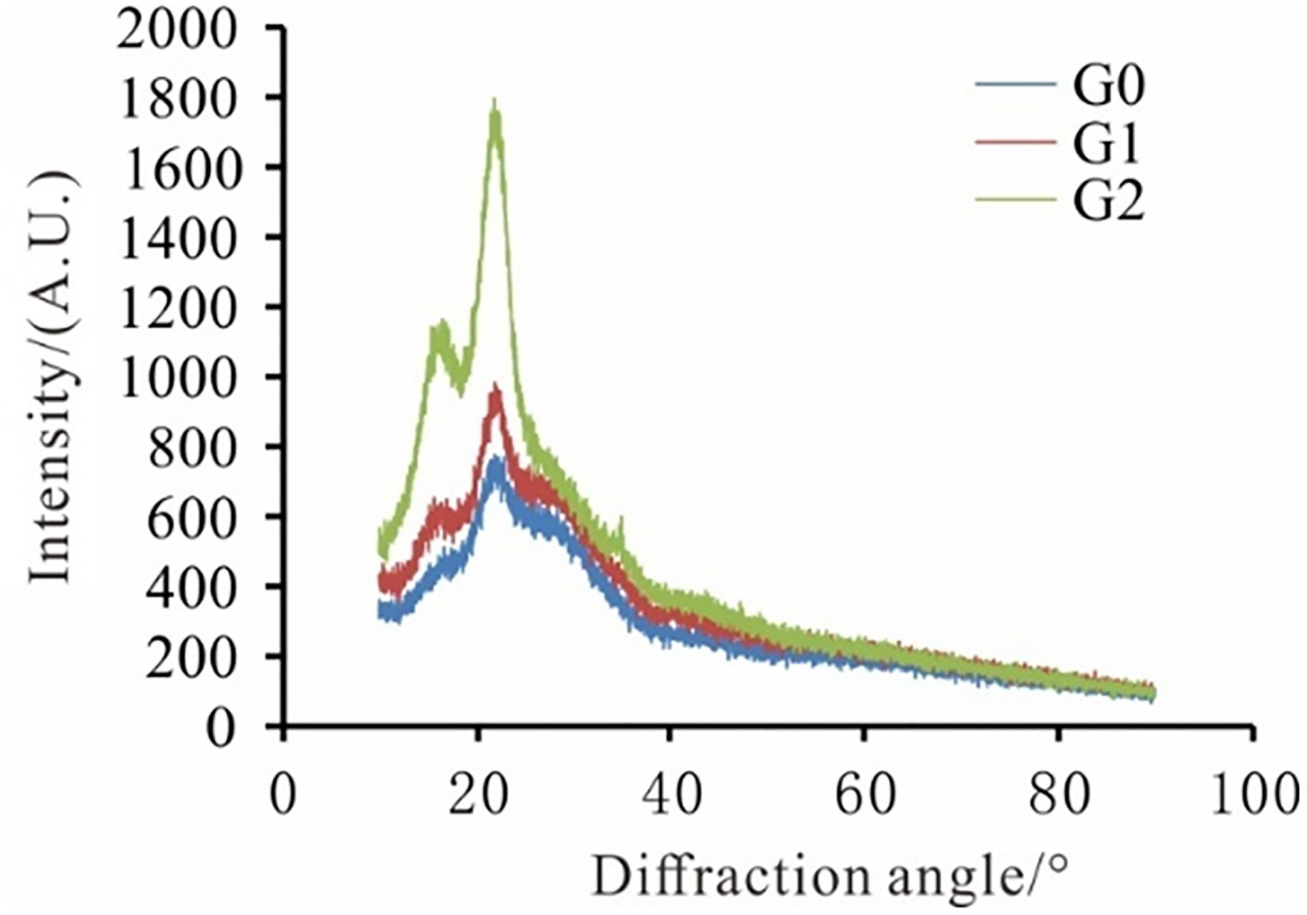
XRD patterns of wheat straw from different fermentation systems.

According to the XRD map, the crystallinity of wheat straw was calculated before and after the fermentation by formula (Fig 7 and Table 10). Moreover, the Segal method was used to calculate the cellulose crystallinity (CrI) [32, 33]:

$${\text{Q}}_{\text{CrI}}=\left(I_{002}-I_{am}\right)/I_{002}\times 100\%$$

Where *Q*_CrI_ denotes the cellulose crystallinity; *I*_002_ stands for the peak intensity of 2θ at about 22°and *I*_am_ represents the peak intensity of 2θ at around 18°.

**Table 10:** XRD crystallinity index of wheat straw.

Samples	*I*_*002*_	*I*_*am*_	*Q*_*CrI*_ */%*
G0	734	469	36.10
G1	928	583	37.18
G2	1720	997	42.03

As can be seen in Table 10, while the crystallinity of G0 was 36.10%, those of G1 and G2 were 37.18% and 42.03% after anaerobic fermentation, respectively. Moreover, compared to the one without pretreatment, the crystallinity of straw increased. This is while the increase in the crystallinity of straw was greater in the mixed fermentation mode. Furthermore, cellulose is mainly composed of two parts, namely, the crystalline region and the amorphous region. The structure of the former was dense, the glucose had no free hydroxyl groups, and accordingly, cellulase could not easily invade into the interior part to play a role in degradation. On the other hand, the structure of the latter, i.e., the amorphous region, was loose and, as a result, was more easily degraded by the microorganisms [33]. Furthermore, the decrease in the content of the cellulose amorphous region during the anaerobic fermentation led to an increase in the relative content of the crystalline region. This, in turn, was demonstrated as an increase in the crystallinity index of the straw, which was the highest in the mixed fermentation group, which was an indication of the advantage of the straw degradation in the combined fermentation process.

## 4. Conclusion

In this study, the characteristics of changes in solid-phase substrates during the co-fermentation of lignite and straw were investigated. The obtained results corroborated the promotion of biomethane production by the mixed fermentation of lignite and straw. Moreover, the ratios the C/H and C/O were found to act complimentarily in the co-fermentation system of straw and lignite after biodegradation. Furthermore, after the fermentation of lignite mixed with straw, the relative content of C-C/C-H bonds was found to be decreased on the surfaces of the lignite and straw. Also, while there was an increase in the aliphatic hydrogen content of the lignite as well as the fat short chains, the fat long chains and the apparent aromaticity decreased. Likewise, while the maturity decreased, there found to be an increase in ratio of the lipid-carbon as well as a tendency to increase of the branched concentration. Finally, while the content of cellulose amorphous zone decreased, the crystallinity of straw increased. Hence, the synergistic fermentation was found to promote the effective degradation of lignite and straw.

## Supporting information

S1 Table Fitting peak position of NMR of coal sample. The nuclear magnetic resonance peak fitting data of the coal sample are derived from the reference literature, which is used as a reference to analyze the peak data of this paper.
(DOCX)

S2 Table Ultimate analyses of the samples. The data were derived from ultimate analysis of coal and straw.
(DOCX)

S1 Fig Electric thermostatic incubator. The picture comes from the constant temperature incubator taken by the laboratory, which is used for the constant temperature culture of anaerobic fermentation experiment.
(TIFF)

S2 Fig Gas fermentation device diagram. The figure is derived from the self-designed and drawn gas production fermentation device, which is used for the combined production of methane from coal and straw.
(TIFF)
