# Supplementary material for: The characteristics of solid-phase substrate during the co-fermentation of lignite and straw
Source: PLoS One. 2023 Jan 26;18(1):e0280890. doi: 10.1371/journal.pone.0280890 (PMC9879535; doi:10.1371/journal.pone.0280890)
Supplement: S1 Table — The nuclear magnetic resonance peak fitting data of the coal sample are derived from the reference literature, which is used as a reference to analyze the peak data of this paper. (DOCX) [file pone.0280890.s001.docx]

Table S1

Fitting peak position of NMR of coal sample

| Functional group | Peak position/ppm | Peak number |
| --- | --- | --- |
| Carbonyl carbon | 190-220 | 13 |
| Carbonyl-c | 165-190 | 12 |
| Oxygen substituted aromatic carbon (phenolic hydroxyl, ether, etc.) | 148-165 | 11 |
| Alkyl substituted aromatic carbon | 137-148 | 10 |
| Bridged aromatic carbon | 129-137 | 9 |
| Protonated aromatic carbon | 100-129 | 8 |
| Carbon in to oxygen in a carbohydrate ring | 75-90 | 7 |
| Aliphatic carbon linked to oxygen | 56-75 | 6 |
| methoxyl group | 50-56 | 5 |
| Quaternary carbon, α-carbon on aromatic ring | 36-50 | 4 |
| Methylene, methylene | 23-36 | 3 |
| Aromatic methyl carbon-CH_3_ | 16-22 | 2 |
| Lipomethyl carbon-CH_3_ | 12-16 | 1 |
