# Supplementary material for: The characteristics of solid-phase substrate during the co-fermentation of lignite and straw
Source: PLoS One. 2023 Jan 26;18(1):e0280890. doi: 10.1371/journal.pone.0280890 (PMC9879535; doi:10.1371/journal.pone.0280890)
Supplement: S2 Table — The data were derived from ultimate analysis of coal and straw. (DOCX) [file pone.0280890.s002.docx]

Table S2

Ultimate analyses of the samples

| Samples | *N*_d_/% | *C*_d_/% | *H*_d_/% | *S*_d_/% | *O*_d_/% |
| --- | --- | --- | --- | --- | --- |
| Wheat straw | 0.51 | 46.22 | 6.03 | 0.16 | 38.70 |
| Lignite | 1.28 | 63.27 | 4.56 | 0.32 | 22.22 |
