# Supplementary figures and images for: The characteristics of solid-phase substrate during the co-fermentation of lignite and straw
Source: PLoS One. 2023 Jan 26;18(1):e0280890. doi: 10.1371/journal.pone.0280890 (PMC9879535; doi:10.1371/journal.pone.0280890)

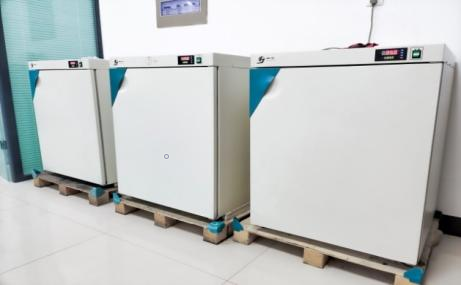

Supplement: S1 Fig — The picture comes from the constant temperature incubator taken by the laboratory, which is used for the constant temperature culture of anaerobic fermentation experiment. (TIFF) [file pone.0280890.s003.tiff]

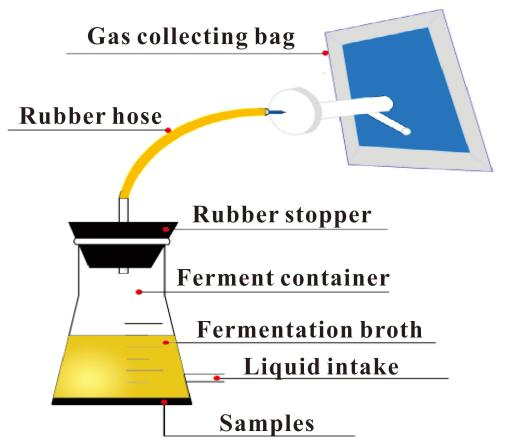

Supplement: S2 Fig — The figure is derived from the self-designed and drawn gas production fermentation device, which is used for the combined production of methane from coal and straw. (TIFF) [file pone.0280890.s004.tiff]
